# Preclinical Evaluation of Oral Urolithin-A for the Treatment of Acute Campylobacteriosis in *Campylobacter jejuni* Infected Microbiota-Depleted IL-10^−/−^ Mice

**DOI:** 10.3390/pathogens10010007

**Published:** 2020-12-23

**Authors:** Soraya Mousavi, Dennis Weschka, Stefan Bereswill, Markus M. Heimesaat

**Affiliations:** Institute of Microbiology, Infectious Diseases and Immunology, Gastrointestinal Microbiology Research Group, Charité-University Medicine Berlin, Corporate Member of Freie Universität Berlin, Humboldt-Universität zu Berlin, and Berlin Institute of Health, 12203 Berlin, Germany; soraya.mousavi@charite.de (S.M.); dennis.weschka@charite.de (D.W.); stefan.bereswill@charite.de (S.B.)

**Keywords:** Urolithin-A, enteropathogenic infection, *Campylobacter jejuni*, immune-modulatory effects, secondary abiotic IL-10^−/−^ mice, campylobacteriosis model, host-pathogen interaction, preclinical intervention study, ellagitannins, lipooligosaccharide

## Abstract

Human campylobacteriosis represents an infectious enteritis syndrome caused by *Campylobacter* species, mostly *Campylobacter jejuni*. Given that *C. jejuni* infections are rising worldwide and antibiotic treatment is usually not indicated, novel treatment options for campylobacteriosis are needed. Urolithin-A constitutes a metabolite produced by the human gut microbiota from ellagitannins and ellagic acids in berries and nuts which have been known for their health-beneficial including anti-inflammatory effects since centuries. Therefore, we investigated potential pathogen-lowering and immunomodulatory effects following oral application of synthetic urolithin-A during acute campylobacteriosis applying perorally *C. jejuni* infected, microbiota-depleted IL-10^−/−^ mice as preclinical inflammation model. On day 6 post infection, urolithin-A treated mice harbored slightly lower pathogen loads in their ileum, but not colon as compared to placebo counterparts. Importantly, urolithin-A treatment resulted in an improved clinical outcome and less pronounced macroscopic and microscopic inflammatory sequelae of infection that were paralleled by less pronounced intestinal pro-inflammatory immune responses which could even be observed systemically. In conclusion, this preclinical murine intervention study provides first evidence that oral urolithin-A application is a promising treatment option for acute *C. jejuni* infection and paves the way for future clinical studies in human campylobacteriosis.

## 1. Introduction

Campylobacteriosis is clinically characterized by acute enteritis upon infection with thermo-tolerant *Campylobacter* species, in most cases *Campylobacter jejuni* [1,2,3,4,5,6]. In particular, the global increase in poultry consumption drives a tremendous rise in human *C. jejuni* infections making campylobacteriosis a serious health problem and economic burden [3,5]. Given that the avian intestinal tract represents the major pathogen reservoir, the symptom-free colonization of livestock such as chickens and turkeys and the subsequent contamination of the meat products during the slaughter process may mount in acute disease and potential post-infectious sequelae in the infected human consumers [3,4,5]. The absence of clinical signs in the *Campylobacter* colonized avian host has been attributed to a non-responsiveness of the birds’ innate immune system to the bacteria. This is in support of the resistance of birds to lipopolysaccharide (LPS) within the cell walls of Gram-negative bacteria including pathogens of the *Campylobacter* clade which produce a truncated variant of LPS termed lipooligosaccharide (LOS) [7]. Results from previous studies suggest that these differences in pathogen recognition might be caused by species-specific variations in the recognition of *Campylobacter* LOS by the specific innate receptor Toll-like-receptor (TLR)-4 [8].

After peroral ingestion, the spiral-shaped motile bacteria multiply in the intestinal lumen of the human host and target enterocytes. Non-flagellated immobile mutants of the pathogen are apathogenic [4,6]. Furthermore, disease induction by *C. jejuni* depends on adhesion of the pathogen to intestinal cells as well as active invasion of enterocytes. In contrast to birds, humans are highly sensitive to LPS/LOS [9] and the human innate immune system, established in the subepithelial tissues, is activated by invading *C. jejuni.* Pathogenic LOS has been shown to be a major virulence factor triggering human campylobacteriosis characterized by bloody diarrhea [10], which may ultimately result in sepsis, particularly in immunocompromised individuals [6]. In turn, innate immune cells such as dendritic cells, neutrophils, macrophages and monocytes are recruited to the infected intestines [4] and produce reactive oxygen species (ROS) thereby promoting intestinal tissue destruction and apoptosis, both histopathologic hallmarks of acute campylobacteriosis [1,2,10]. The essential role of *C. jejuni*-LOS in human campylobacteriosis is further underlined by post-infectious sequelae such as Guillain–Barré syndrome or reactive arthritis which have been shown to be significantly associated with sialylated LOS of the infecting *C. jejuni* strain, and additionally with the severity of the preceding gastrointestinal disease [11]. The role of *C. jejuni*-LOS as a master regulator during human campylobacteriosis was finally confirmed at the molecular level, indicating that LOS induced innate immune activation triggers apoptosis and sodium malabsorption [10]. In consequence, LOS induced innate immune responses provide a useful target for the development of novel pharmaceutical intervention strategies in order to dampen *C. jejuni* induced immunopathogenesis. However, this necessitates the development of convenient and reliable experimental in vivo infection and inflammation models which has been complicated by the effective physiological colonization resistance of conventional laboratory mice against *C. jejuni* [12] and furthermore, by the approximately 10,000-fold less responsiveness of mice to LOS when compared to humans [13]. These limitations were solved by generating microbiota-depleted mice following antibiotic treatment which are sensitized to LOS by deficiencies in genes for interleukin (IL) 10 or single-Ig-interleukin-1 related receptor (SIGIRR) [7,14,15]. Upon *C. jejuni* infection, these mice develop severe intestinal inflammation and clinical signs of human campylobacteriosis, which also holds true for intestinal histopathological changes characterized by innate immune cell accumulation, apoptosis and tissue destruction within the infected intestines [2,16,17,18]. The role of LOS in campylobacteriosis was finally confirmed by the amelioration of *C. jejuni* induced enterocolitis in mice lacking TLR4 [7]. Given that microbiota-depleted and LOS sensitized IL-10^−/−^ mice did not develop campylobacteriosis upon infection with non-motile, flagella-deficient *C. jejuni* mutants [19], these mice were further used as a convenient model for studying *C. jejuni* virulence factors triggering acute campylobacteriosis [7], including proteases such as PepP [20] and HtrA [21,22]. This campylobacteriosis model was further successfully used to evaluate disease-alleviating properties of distinct molecules such as vitamin C [23], vitamin D [24], carvacrol [25], and the neuropeptides PACAP [26] and NAP [27] in acute *C. jejuni* induced enterocolitis [7]. 

To provide novel treatment options for campylobacteriosis in a preclinical setting, we further searched for a pharmaceutically well-documented non-toxic molecule, which combines potent anti-inflammatory and anti-oxidant effects with rather low anti-microbial activities to avoid the emergence of pathogenic resistance and intestinal dysbiosis. In fact, urolithin-A constitutes a promising candidate fulfilling all these prerequisites. The molecule is produced by the human gut microbiota from dietary plant-derived ellagitannins and ellagic acid present in berries, pomegranate, and nuts, which have been known for their anti-inflammatory effects since centuries [28,29,30,31]. Treatment studies revealed that urolithin-A and its precursors ameliorate chemically induced colitis in rodents [32] and protect humans from both acute and chronic inflammatory diseases [33]. Modes of action by urolithin-A include direct oxygen radical scavenging properties [34]. It further dampens LPS/LOS induced innate immune responses [31,35,36,37] and ROS production by inhibition of myeloperoxidases (MPO) / lactoperoxidases (LPO) and the nuclear-factor-kappa-B (NFκB) subunit translocation to the nucleus even at low concentrations [38]. 

Therefore, these scientifically confirmed health-beneficial effects prompted us to evaluate urolithin-A as a potential novel treatment option for acute campylobacteriosis in *C. jejuni* infected microbiota-depleted IL-10^−/−^ mice. The major aim of our study was to test whether synthetic urolithin-A applied via the oral route in drinking water at low concentrations (in the µM range) ameliorates pathogen-induced intestinal and systemic inflammation. 

## 2. Results

### 2.1. Gastrointestinal Pathogen Loads following Urolithin-A Treatment of C. jejuni Infected IL-10^−/−^ Mice

**Figure 1:**
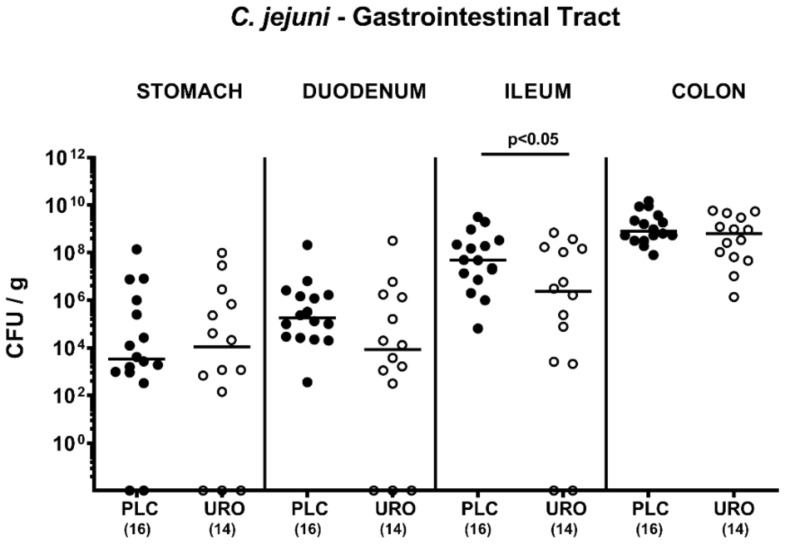
Gastrointestinal pathogen numbers upon urolithin-A treatment of *C. jejuni* infected IL10^−/−^ mice. On day (0) and d1, microbiota depleted IL-10^−/−^ mice were orally infected with *C. jejuni* strain 81-176 and treated with urolithin-A (URO, open circles) or placebo (PLC, closed circles) via the drinking water starting on d2 post infection. On d6, the gastrointestinal pathogen loads were determined by culture (expressed as colony forming units per g luminal content, CFU/g). Medians (black bars), significance levels (*p* values) determined by the Mann­–Whitney U test and total numbers of included animals (in parentheses) are given. Data were pooled from three independent experiments.

Microbiota depleted IL-10^−/−^ mice were perorally infected with 10^9^ colony forming units (CFU) *C. jejuni* strain 81-176 on days 0 and 1 and subjected to either urolithin-A or placebo treatment via the drinking water as initiated on day 2 p.i. Our cultural analyses revealed that on days 2, 3 and 4 post infection (p.i.), fecal *C. jejuni* numbers were lower in the urolithin-A as compared to the placebo cohort (*p* < 0.01–0.001), whereas later-on, comparably high median pathogen loads of approximately 10^9^ CFU per g fecal sample could be determined (not significant (n.s.); Supplementary Figure S1). On day 6 p.i., we assessed the pathogen burdens in defined compartments within the gastrointestinal tract and found comparable luminal *C. jejuni* cell numbers in the stomach, duodenum and colon of urolithin-A and placebo treated mice (n.s.; Figure 1). In the ileum, however, median *C. jejuni* loads were almost two log orders of magnitude lower in the former versus the latter (*p* < 0.05; Figure 1). Of note, two and three mice from the urolithin-A cohort had expelled the *C. jejuni* bacteria from the ileal and duodenal lumen until day 6 p.i., respectively, whereas all placebo counterparts were *C. jejuni* culture-positive (Figure 1). Hence, ileal pathogen burdens were lower following urolithin-A versus placebo application to *C. jejuni* infected IL-10^−/−^ mice.

### 2.2. Clinical Outcome Upon Urolithin-A Treatment of C. jejuni Infected IL-10^−/−^ Mice

**Figure 2:**
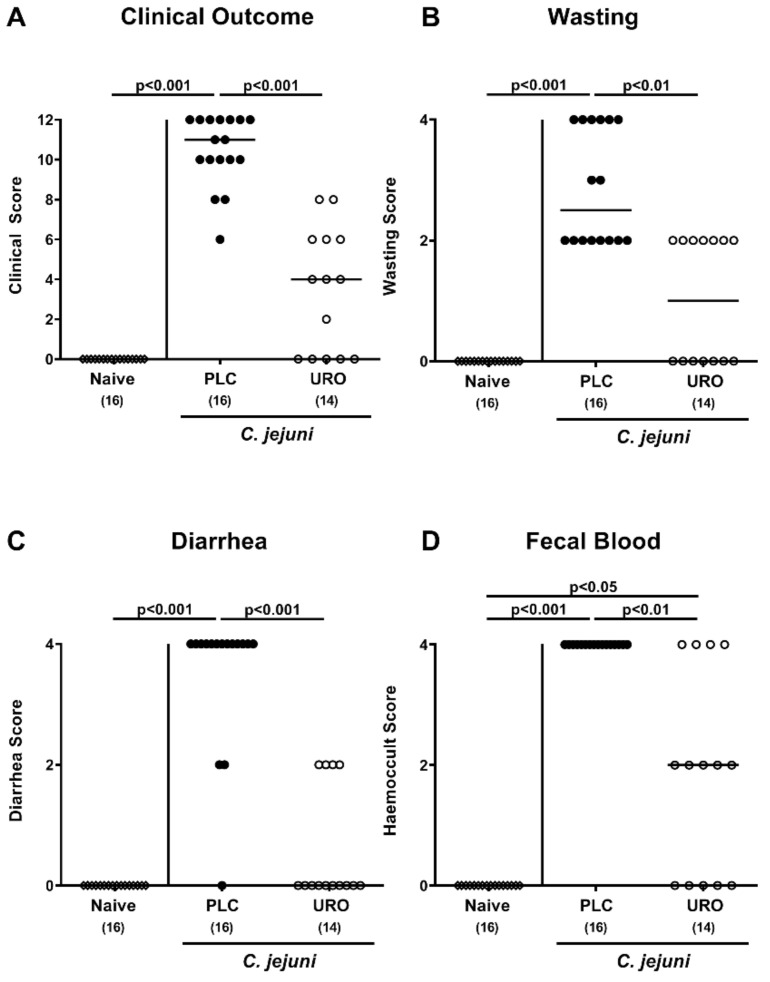
Clinical outcome upon urolithin-A application to *C. jejuni* infected IL-10^−/−^ mice. Following *C. jejuni* strain 81-176 infection on days 0 and 1, microbiota depleted IL-10^−/−^ mice were treated with urolithin-A (URO, open circles) or placebo (PLC, closed circles) via the drinking water starting on d2 post infection. Prior and at distinct time points post infection, the (**A**) overall clinical outcome was quantified in each mouse by standardized clinical scores assessing in particular (**B**) wasting, (**C**) stool consistency (diarrhea) and (**D**) fecal blood. Naive mice were used as negative controls (open diamonds). Medians (black bars), significance levels (*p* values) calculated by the ANOVA test with Tukey post-correction or by the Kruskal–Wallis test and Dunn’s post-correction and the total numbers of included animals (in parentheses) are given. Data were pooled from three independent experiments.

Next, we surveyed the clinical course in *C. jejuni* infected mice during urolithin-A treatment. When applying clinical scores for typical clinical signs of acute campylobacteriosis including wasting and bloody diarrhea, mice from the urolithin-A cohort displayed lower clinical scores as compared to placebo counterparts as early as day 2 p.i. (*p* < 0.01–0.001; Supplementary Figure S2). At the end of the observation period on day 6 p.i., when placebo mice suffered from severe *C. jejuni* induced disease, urolithin-A treated animals exhibited less pronounced clinical signs (*p* < 0.01–0.001; Figure 2). Notably, 35.7% of mice from the urolithin-A cohort were clinically uncompromised and were lacking any signs of disease (Figure 2). Hence, urolithin-A treatment led to a better clinical outcome in *C. jejuni* infected IL-10^−/−^ mice. 

### 2.3. Macroscopic and Microscopic Inflammatory Outcomes Upon Urolithin-A Treatment of C. jejuni Infected IL-10^−/−^ Mice

**Figure 3:**
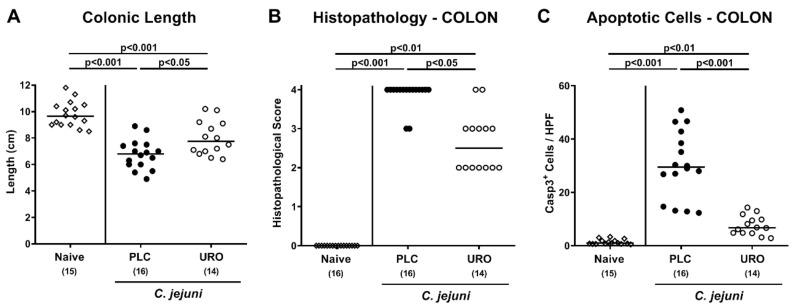
Macroscopic and microscopic inflammatory outcomes upon urolithin-A application to *C. jejuni* infected IL-10^−/−^ mice. Following *C. jejuni* strain 81-176 infection on days 0 and 1, microbiota depleted IL-10^−/−^ mice were treated with urolithin-A (URO, open circles) or placebo (PLC, closed circles) via the drinking water starting on d2 post infection. On d6, (**A**) the colonic lengths were measured and (**B**) the histopathological changes in large intestinal ex vivo biopsies quantified using standardized histopathology scores. (**C**) Furthermore, the average numbers of apoptotic colonic epithelial cells (positive for cleaved caspase3, Casp3^+^) were determined microscopically from six high power fields (HPF, 400 × magnification) per mouse applying in situ immunohistochemical analysis of large intestinal paraffin sections. Naive mice were used as negative controls (open diamonds). Medians (black bars), significance levels (*p* values) calculated by the ANOVA test with Tukey post-correction or by the Kruskal–Wallis test and Dunn’s post-correction and the total numbers of included animals (in parentheses) are given. Data were pooled from three independent experiments.

Since significant shrinkage of the affected intestine can be observed during intestinal inflammation [39], we measured the large intestinal lengths of each mouse after sacrifice. On day 6 p.i., mice from both the urolithin-A and placebo groups displayed shorter colons when compared to naive animals (*p* < 0.001), but with longer colonic lengths measured in the former as compared to the latter (*p* < 0.05; Figure 3A), indicative of less pronounced gross *C. jejuni* induced large intestinal inflammation upon urolithin-A treatment. We further quantified the severity of colonic inflammation microscopically by histopathology scoring of hematoxylin and eosin stained colonic paraffin sections. *C. jejuni* infection of mice from the placebo cohort resulted in severe histopathological changes observed in colonic tissue samples (*p* < 0.001 versus naive), which were, however, less pronounced in urolithin-A versus placebo treated mice on day 6 p.i. (*p* < 0.05; Figure 3B). We additionally assessed pathogen-induced apoptotic responses in colonic epithelial cells after immunohistochemical staining of colonic samples. *C. jejuni* infection resulted in increased apoptotic epithelial cell counts (*p* < 0.01–0.001), but with lower numbers in urolithin-A as compared to placebo treated mice on day 6 p.i. (*p* < 0.001; Figure 3C). Hence, urolithin-A treatment of IL-10^−/−^ mice resulted in less pronounced macroscopic as well as microscopic inflammatory responses following *C. jejuni* infection.

### 2.4. Colonic Pro-Inflammatory Immune Responses Upon Urolithin-A Treatment of C. jejuni Infected IL-10^−/−^ Mice

**Figure 4:**
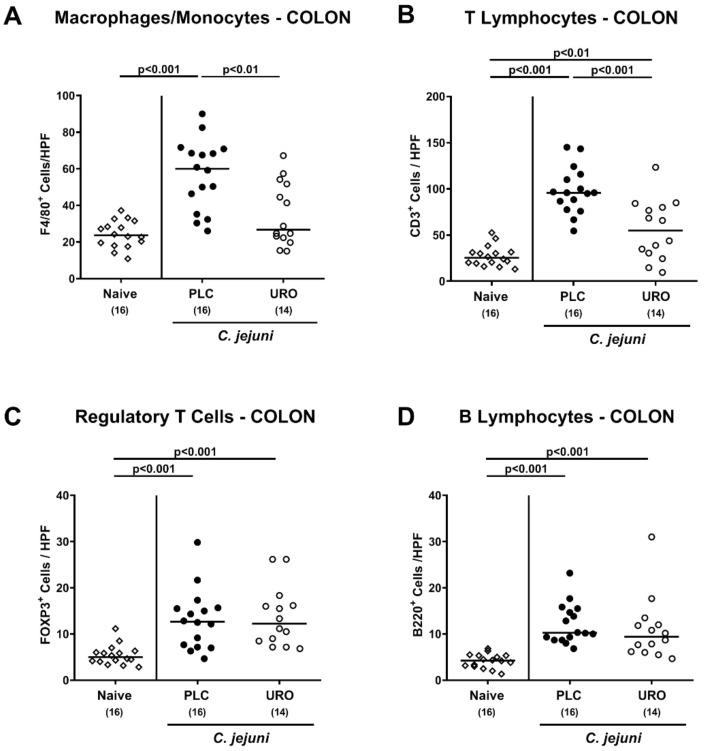
Colonic immune cell responses upon urolithin-A application to *C. jejuni* infected IL-10^−/−^ mice. Following *C. jejuni* strain 81-176 infection on days 0 and 1, microbiota depleted IL-10^−/−^ mice were treated with urolithin-A (URO, open circles) or placebo (PLC, closed circles) via the drinking water starting on d2 post infection. On d6, the average numbers of (**A**) F4/80^+^ macrophages and monocytes) (**B**) CD3^+^ T lymphocytes), (**C**) FOXP3^+^ regulatory T cells and (**D**) B220^+^ B lymphocytes were determined microscopically from six high power fields (HPF, 400 × magnification) per animal in immunohistochemically stained large intestinal paraffin sections. Naive mice were used as negative controls (open diamonds). Medians (black bars), significance levels (*p* values) calculated by the ANOVA test with Tukey post-correction or by the Kruskal–Wallis test and Dunn’s post-correction and the total numbers of included animals (in parentheses) are given. Data were pooled from three independent experiments.

In order to dissect potential immunomodulatory properties of urolithin-A in *C. jejuni* infected mice in more detail, we quantified distinct immune cell populations in the colonic mucosa and lamina propria applying in situ immunohistochemistry. On day 6 p.i., colonic numbers of macrophages and monocytes and of T lymphocytes were lower in urolithin-A versus placebo treated mice (*p* < 0.01 and *p* < 0.001, respectively; Figure 4A,B), whereas *C. jejuni* induced increases in regulatory T cells and B lymphocytes within the large intestines were comparable in both treatment cohorts (*p* < 0.001 versus naive; Figure 4C,D).

**Figure 5:**
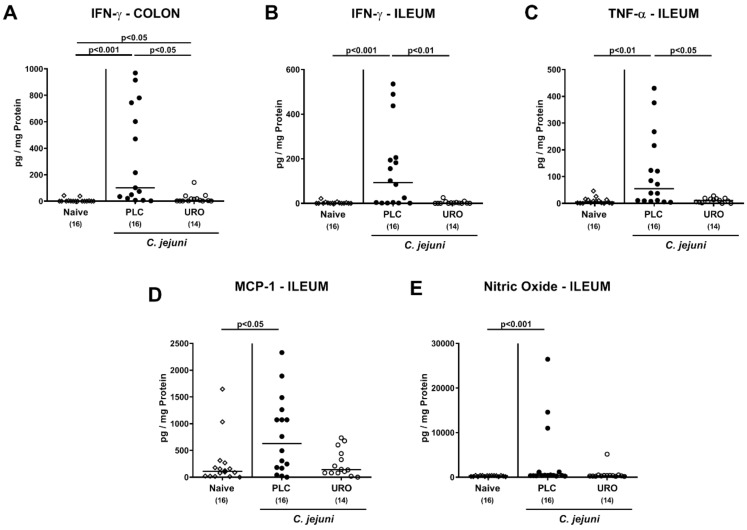
Intestinal pro-inflammatory mediator concentrations upon urolithin-A application to *C. jejuni* infected IL-10^−/−^ mice. Following *C. jejuni* strain 81-176 infection on days 0 and 1, microbiota depleted IL-10^−/−^ mice were treated with urolithin-A (URO, open circles) or placebo (PLC, closed circles) via the drinking water starting on d2 post infection. On d6 post infection, (**A**,**B**) IFN-γ secretion as well as (**C**) TNF-α, (**D**) MCP-1 and (**E**) nitric oxide concentrations were assessed in ex vivo biopsies derived from the (**A**) colon and (**B**–**E**) ileum. Naive mice were used as negative controls (open diamonds). Medians (black bars), significance levels (*p* values) calculated by the ANOVA test with Tukey post-correction or by the Kruskal–Wallis test and Dunn’s post-correction and the total numbers of included animals (in parentheses) are given. Data were pooled from three independent experiments.

We further assessed intestinal pro-inflammatory mediator secretion and determined lower interferon-γ (IFN-γ) concentration in both colonic and ileal explants taken from urolithin-A versus placebo treated mice on day 6 p.i. (*p* < 0.05 and *p* < 0.001, respectively; Figure 5A,B) which also held true for ileal tumor necrosis factor-α (TNF-α) protein levels (*p* < 0.05; Figure 5C). Furthermore, increased monocyte chemoattractant protein-1 (MCP-1) and nitric oxide concentrations were assessed in the ileum of placebo, but not urolithin-A treated *C. jejuni* infected mice (*p* < 0.05 and *p* < 0.001, respectively; Figure 5D,E). Hence, urolithin-A ameliorated *C. jejuni* induced pro-inflammatory immune responses in the intestinal tract of infected IL-10^−/−^ mice.

### 2.5. Extra-Intestinal Pro-Inflammatory Mediators Upon Urolithin-A Treatment of C. jejuni Infected IL-10^−/−^ Mice

**Figure 6:**
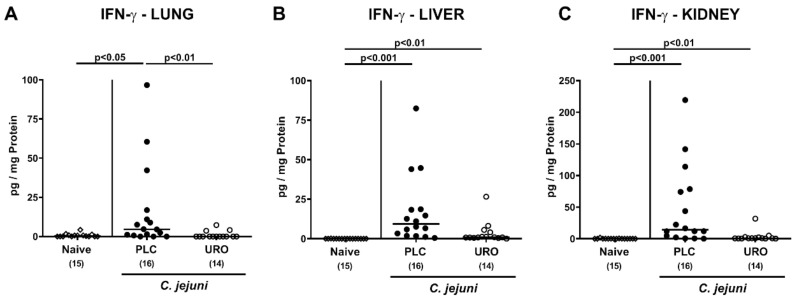
Extra-intestinal IFN-γ concentrations upon urolithin-A application to *C. jejuni* infected IL-10^−/−^ mice. Following *C. jejuni* strain 81-176 infection on days 0 and 1, microbiota depleted IL-10^−/−^ mice were treated with urolithin-A (URO, open circles) or placebo (PLC, closed circles) via the drinking water starting on d2 post infection. On d6 post infection, extra-intestinal IFN-γ concentrations were measured in ex vivo biopsies derived from the (**A**) lungs, (**B**) liver and (**C**) kidneys. Naive mice were used as negative controls (open diamonds). Medians (black bars), significance levels (*p* values) calculated by the ANOVA test with Tukey post-correction or by the Kruskal–Wallis test and Dunn’s post-correction and the total numbers of included animals (in parentheses) are given. Data were pooled from three independent experiments.

We next asked whether the immunomodulatory properties of exogenous urolithin-A might also be observed in extra-intestinal tissues. On day 6 p.i., increased IFN-γ concentrations were determined in lungs from placebo but not urolithin-A treated mice (*p* < 0.01; Figure 6A). *C. jejuni* infection was further accompanied by elevated IFN-γ concentrations in liver and kidney samples taken from mice of both cohorts (*p* < 0.01–0.001; Figure 6B,C), but with a trend towards lower IFN-γ concentrations in urolithin-A versus placebo treated mice (n.s. due to high standard deviations; Figure 6B,C). 

**Figure 7:**
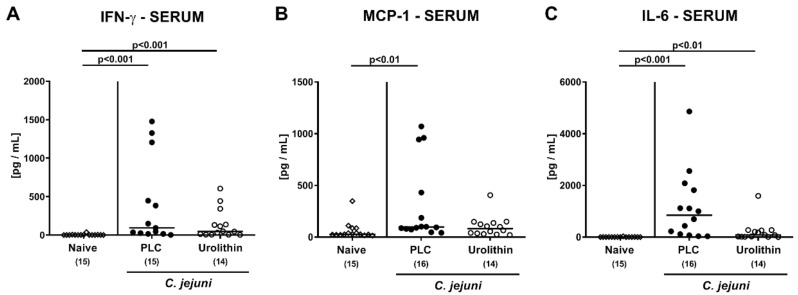
Systemic pro-inflammatory cytokine concentrations upon urolithin-A application to *C. jejuni* infected IL-10^−/−^ mice. Following *C. jejuni* strain 81-176 infection on days 0 and 1, microbiota depleted IL-10^−/−^ mice were treated with urolithin-A (URO, open circles) or placebo (PLC, closed circles) via the drinking water starting on d2 post infection. On d6 post infection, (**A**) IFN-γ, (**B**) MCP-1 and (**C**) IL-6 concentrations were measured in serum samples. Medians (black bars), significance levels (*p* values) calculated by the ANOVA test with Tukey post-correction or by the Kruskal–Wallis test and Dunn’s post-correction and the total numbers of included animals (in parentheses) are given. Data were pooled from three independent experiments.

We further assessed systemic pro-inflammatory mediator secretion upon urolithin-A application. On day 6 p.i., comparable IFN-γ and IL-6 concentrations were determined in serum samples derived from mice of both treatment groups (n.s.; Figure 7A,C), but with a trend towards lower IL-6 levels in urolithin-A versus placebo treated animals (n.s. due to high standard deviations; Figure 7C). In mice from the placebo, but not the urolithin-A cohort, however, increased MCP-1 serum concentrations were detected upon sacrifice (*p* < 0.01 versus naive; Figure 7B). Hence, the immune response-dampening effects of external urolithin-A could also be observed beyond the intestinal tract in extra-intestinal compartments of *C. jejuni* infected IL-10^−/−^ mice.

## 3. Discussion

The work presented here is a first preclinical intervention study evaluating the disease-alleviating efficacy of oral urolithin-A treatment in murine campylobacteriosis and reveals that the anti-inflammatory properties of the ellagitannin derivative could ameliorate *C. jejuni* infection locally in the intestinal tract and even beyond (i.e., in distinct extra-intestinal tissues sites as well as systemically). Peroral application of urolithin-A versus placebo to *C. jejuni* infected microbiota depleted IL-10^−/−^ mice resulted in: (1) improved clinical conditions observed as early as 24 hours following initiation of treatment; (2) less distinct macroscopic intestinal sequelae as indicated by less colonic shrinkage; (3) less pronounced microscopic inflammatory changes, as indicated by less colonic histopathology and apoptosis; (4) lower abundance of macrophages, monocytes and T lymphocytes in the colonic mucosa and lamina propria on day 6 p.i.; (5) reduced pro-inflammatory mediator secretion in the intestinal tract and in extra-intestinal as well as systemic compartments. In line with the absence of any clinical signs in more than one third of infected mice, the significant reduction in wasting, fecal blood, and diarrhea in the treated versus control animals demonstrates that the effects of urolithin-A concern all clinical aspects of *C. jejuni* induced disease.

The fact that urolithin-A exerted all these beneficial properties without lowering the *C. jejuni* loads in the colon is of importance given that promotion of *C. jejuni* resistance against the drug is not relevant for successful treatment. The biological relevance of the reduction in *C. jejuni* loads in the ilea of urolithin-A versus placebo treated IL-10^−/−^ mice is put in perspective by the low solubility of urolithin-A at concentrations above those applied here. While the antimicrobial activity of urolithin-A against *C. jejuni* has not been investigated so far, results from previous studies demonstrated that ellagitannins and phlorotannins can inhibit *C. jejuni* growth in vitro [29,40,41].

Most importantly, urolithin-A treatment of acute murine campylobacteriosis resulted in less pronounced macroscopic as well as microscopic inflammatory responses upon *C. jejuni* infection. The better clinical outcome of the animals could be attributed to decreased intestinal inflammation as indicated by lower histopathological scores in the treated versus control animals. Significantly, reduced intestinal inflammatory cellular infiltrates indicate that urolithin-A dampens the innate immune responses during the early phase of *C. jejuni* infection. Given that the disease is characterized by infiltration of the intestinal tissues by both innate and adaptive immune cell subsets, the alleviation of this histopathological hallmark of human campylobacteriosis by urolithin-A treatment is essential for the bench-to-bedside transfer of the obtained results. In line with the potent inhibition of inflammatory infiltrates, the anti-inflammatory urolithin-A effects were further confirmed by decreased numbers of macrophages and T lymphocytes in the intestines of treated as compared to control animals. Furthermore, reduced numbers of apoptotic cells within the colon of verum versus placebo treated mice point towards cell-protective effects of urolithin-A in the intestinal tract during *C. jejuni* infection. In support, both urolithins and the ellagitannins were shown to suppress apoptosis in inflammation [31,42,43]. Anti-apoptotic effects of urolithin-A are also in agreement with the finding that urolithin-A improved chemically induced colitis by activation of nuclear factor erythroid 2–related factor 2 (Nrf 2) dependent intestinal epithelial barrier functions and aryl-hydrocarbon receptor induced anti-inflammatory and anti-oxidative cellular pathways [44]. Similar cell-protective mechanisms have been recently reported for urolithin derivatives [45], and those intracellular pathways include protection against oxidative stress caused by ROS and immune activators which are produced by innate immune cells in response to *C. jejuni*-LOS and other bacterial factors triggering inflammation. The lowered nitric oxide concentrations in the ileum of urolithin-A treated mice observed in this study are well in line with the direct ROS scavenging functions of urolithin-A and its capacity to inhibit ROS production by innate immune cells via blockage of MPO, LPO and inducible nitric oxide synthase (iNOS) activities [31,34,38]. Furthermore, the inhibition of LPS mediated activation of innate immune cells by urolithin-A has been well investigated in vitro, and this feature validated the analysis of the molecule as a potential candidate for novel pharmaceutical interventions against campylobacteriosis in the present study [36,46,47]. The multifactorial mechanisms by which urolithin-A exerts its anti-inflammatory activities are completed by the inhibition of the central immune activator NFκB in innate immune cells in vitro [35]. Given that *C. jejuni* induced pathologies include the activation of the mammalian target of rapamycin (mTOR) and were ameliorated by rapamycin treatment in IL-10^−/−^ mice [48], it is noteworthy that urolithin-A was reported to downregulate mTOR dependent pathways in cancer cells [49]. Taken together, these multi-faceted beneficial effects of urolithin-A in ameliorating acute murine campylobacteriosis can be attributed to a concerted action of cell-protective and anti-inflammatory mechanisms resulting in dampened LPS/LOS induced inflammatory responses of the innate immune system upon intestinal *C. jejuni* infection.

The anti-inflammatory effects of urolithin-A are further supported by the reduction in soluble pro-inflammatory mediators including MCP-1, TNF-α and IFN-γ in the intestinal tissues of treated versus control mice. This protects the animals from systemic inflammation, as is further supported by the reduced extra-intestinal and even systemic pro-inflammatory mediator secretions in the lungs, the kidneys and the liver as well as in the serum of urolithin-A versus placebo-treated mice, respectively. In support, ellagitannins, coumarins and urolithin-A reduced LPS-induced lung inflammation and osteoarthritis in respective murine models of systemic inflammation [42,45,50]. However, systemic effects of urolithin-A are controversially discussed mainly because upon resorption enzymatic modification of urolithin-A leads to less active glucuronidated and sulfonated variants [29,36]. On the other hand, continuous intake via drinking water was shown to elevate systemic urolithin-A concentrations in mice and the resulting anti-oxidant capacity of murine sera contributed to the healing of experimentally induced paw edema [51]. Furthermore, analysis of systemic inflammation in LPS treated rats revealed that de-glucuronidation of urolithin is induced at inflamed tissue sites [52].

The transfer of the here-obtained preclinical results to defined treatment measures of human campylobacteriosis are supported by the fact that anti-inflammatory and gut epithelial barrier strengthening functions of urolithins and ellagitannin precursors have been confirmed in humans recently [42]. Furthermore, urolithin-A has been shown to exert potent gut protective functions [43], and safety evaluation in humans and rats revealed the complete absence of unwanted side effects [53]. Hence, patients suffering from campylobacteriosis may benefit from the ingestion of urolithin precursors in walnuts and pomegranate juice, which are cheap and easily accessible. It is noteworthy that the ellagitannins present in the food components also exhibit a wide range of biological activities including anti-inflammatory, anti-thrombotic, anti-atherogenic, and anti-angiogenic properties in the absence of unwanted side effects [29,30,31,33]. 

## 4. Materials and Methods

### 4.1. Ethical Statement

Animal welfare was monitored daily by assessment of clinical conditions. The experiments in mice were carried out according to the European Guidelines for animal welfare (2010/63/EU) and were approved by the commission for animal experiments located at the “Landesamt für Gesundheit und Soziales” (LaGeSo, Berlin; registration number G0104/19, approved on 15 July 2019).

### 4.2. Microbiota Depleted IL-10^−/−^ Mice

All mice were bred in the Forschungsinstitute für Experimentelle Medizin, Charité—University Medicine Berlin (Germany). In the age of six weeks IL-10^−/−^ mice in the C57BL/6j background were kept in cages with filter tops in a semi-barrier setting under standard conditions (55 ± 15% air humidity) at room temperature (22–24 °C) with a 12-hour light and a 12-hour dark cycle. The animals had free access to autoclaved water and standard chow consisting of food pellets (ssniff R/M-H, V1534-300, Sniff, Soest, Germany). By the age of 7 weeks, female and male mice were transferred to sterile cages (maximum of 4 animals per cage) and treated with an antibiotic cocktail consisting of ampicillin plus sulbactam (1 g/L; Dr. Friedrich Eberth Arzneimittel, Ursensollen, Germany), vancomycin (500 mg/L; Hikma Pharmaceuticals, London, UK), ciprofloxacin (200 mg/L; Fresenius Kabi, Bad Homburg, Germany), imipenem (250 mg/L; Fresenius Kabi, Bad Homburg, Germany) and metronidazole (1 g/L; B. Braun, Melsungen, Germany) for eight weeks (via the drinking water, ad libitum). This broad-spectrum antibiotic treatment led to a virtual depletion of the commensal intestinal microbiota, as shown earlier [39,54]. Microbiota-depleted mice received autoclaved food and tap water. To minimize the risk of contaminations, the animals were continuously kept and handled under strict aseptic conditions. Three days prior to *C. jejuni* infection, the antibiotic cocktail was replaced by autoclaved tap water to assure washout of the antibiotics.

### 4.3. Campylobacter jejuni Infection

Age- and sex-matched secondary abiotic IL-10^−/−^ mice (4-month-old litter mates) were infected perorally with 10^9^ CFU of the *C. jejuni* strain 81-176 on days 0 and 1 by oral gavage. The *C. jejuni* bacteria originating from a standardized frozen stock were first grown on selective Karmali agar plates and on columbia agar with 5% sheep blood. Both solid growth media were purchased from Oxoid (Wesel, Germany). 

### 4.4. Urolithin-A Treatment

Treatment of mice with urolithin-A (Sigma-Aldrich, Munich, Germany) in drinking water was performed with a daily dose of 0.114 mg urolithin-A per kg body weight per day. This was achieved by dissolving the compound in autoclaved tap water (ad libitum) to a final concentration of 22.8 mg/L. Mice received access to the urolithin-A solution from two days after the first *C. jejuni* infection. The placebo control mice received autoclaved tap water instead.

### 4.5. Gastrointestinal C. jejuni Loads and Bacterial Translocation

*C. jejuni* concentrations were determined in luminal samples taken from the stomach, duodenum, ileum, colon and in feces over time p.i. as well as in homogenates of ex vivo biopsies taken from mesenteric lymph nodes (MLN), spleens, livers, kidneys and cardiac blood upon necropsy by culture, as described previously [12,55]. The intraluminal gastrointestinal samples and respective ex vivo biopsies were homogenized in sterile phosphate buffered saline (PBS; Thermo Fisher Scientific, Waltham, MA, USA), and serial dilutions were plated onto columbia agar (with 5% sheep blood) and Karmali agar (both from Oxoid, Wesel, Germany). Bacterial growth was monitored after incubation of solid media in a microaerophilic atmosphere for at least 48 hours (37 °C). Cardiac blood (0.2 mL) was immediately streaked onto Karmali agar plates. The detection limit of viable *C. jejuni* was 100 CFU per gram analyzed sample.

### 4.6. Clinical Outcome

The clinical outcome of campylobacteriosis in mice was assessed immediately before and after infection once daily by using a standardized cumulative clinical score (maximum 12 points) as described earlier [22]. The clinical score addressed the clinical aspect/wasting (0: normal; 1: ruffled fur; 2: less locomotion; 3: isolation; 4: severely compromised locomotion, pre-final aspect) and the abundance of blood in feces (0: no blood; 2: microscopic detection of blood by the Guajac method using Haemoccult, Beckman Coulter/PCD, Krefeld, Germany; 4: macroscopic blood visible) as well as the stool consistency (0: formed feces; 2: pasty feces; 4: liquid feces).

### 4.7. Sampling Procedures

On day 6 p.i., mice were sacrificed by CO_2_ asphyxiation and colonic lengths were measured with a ruler. From each animal cardiac blood, ex vivo biopsies of lungs, livers, kidneys, MLN, ileum and colon as well as luminal samples from stomach, duodenum, ileum, and colon were derived under aseptic conditions.

### 4.8. Histopathology

Histopathological analyses were performed in colonic ex vivo biopsies (immediately fixed in 5% formalin, embedded in paraffin, and stained with hematoxylin and eosin) as reported previously [56]. Briefly, gross histopathological changes assessed microscopically in 100 times magnified tissue sections of 5 μm thickness were scored by an independent investigator using an established histopathological scoring system: Score 1: minimal inflammatory cell infiltrates in the mucosa with intact epithelium. Score 2: mild inflammatory cell infiltrates in the mucosa and submucosa with mild hyperplasia and mild goblet cell loss. Score 3: moderate inflammatory cell infiltrates in the mucosa with moderate goblet cell loss. Score 4: marked inflammatory cell infiltration into in the mucosa and submucosa with marked goblet cell loss, multiple crypt abscesses and crypt loss.

### 4.9. In Situ Immunohistochemistry

Quantitative in situ immunohistochemical analyses were performed by counting positively stained cells by blinded independent investigators microscopically in at least six high power fields (HPF, 0.287 mm^2^, 400 × magnification) of tissue sections (see above) of intestinal ex vivo biopsies as recently reported [20,57]. In brief, detection of apoptotic epithelial cells, macrophages and monocytes, T lymphocytes, regulatory T cells and B lymphocytes, 5 µm thin colonic paraffin sections were stained with primary antibodies directed against cleaved caspase3 (Asp175, Cell Signaling, Beverly, MA, USA, 1:200), F4/80 (no. 14-4801, clone BM8, eBioscience, San Diego, CA, USA, 1:50), CD3 (no. N1580, Dako, 1:10), FOXP3 (clone FJK-165, no. 14-5773, eBioscience, 1:100) and B220 (no. 14-0452-81, eBioscience; 1:200), respectively. 

### 4.10. Measurement of Pro-Inflammatory Mediators

Pro-inflammatory mediator and nitric oxide secretion in serum samples and in ex vivo biopsies were measured with the Mouse Inflammation Cytometric Bead Assay (CBA; BD Biosciences, Heidelberg, Germany) in a BD FACSCanto II flow cytometer (BD Biosciences, Heidelberg, Germany) and by the Griess reaction [39,58], respectively. Measurements were undertaken in supernatants of ex vivo biopsies that had been incubated in 24-flat-bottom well culture plates (Thermo Fisher Scientific, Waltham, MA, USA) containing 500 μL serum-free RPMI 1640 medium (Thermo Fisher Scientific, Waltham, MA, USA) after incubation at 37 °C for 18 hours. Respective culture supernatants and serum samples were tested for IFN-γ, TNF-α, MCP-1 and IL-6. Briefly, the biopsies obtained from the colon and ileum were longitudinally cut in strips of approximately 1 cm^2^ and washed in PBS (Thermo Fisher Scientific, Waltham, MA, USA). The extra-intestinal ex vivo biopsies were taken from one lung, the liver (approximately 1 cm^3^) and one-half of a kidney (after longitudinal cut). The biopsies were supplemented with penicillin (100 µg/mL) and streptomycin (100 µg/mL; Biochrom, Berlin, Germany). 

### 4.11. Statistical Analyses

The medians and significance levels were calculated by using GraphPad Prism v8, USA. For pairwise comparisons of normally and not normally distributed data, the Student’s *t* test and the Mann–Whitney test were applied, respectively. Furthermore, for multiple comparisons we used the one-sided ANOVA with Tukey post-correction (for normally distributed data) and the Kruskal–Wallis test with Dunn’s post-correction (for not normally distributed data). After definite outliers were removed by identification with the Grubb’s test (α = 0.001), two-sided probability (*p*) values ≤ 0.05 were considered significant. We used pooled data from three independent experiments for all the analyses.

## 5. Conclusions

In conclusion, synthetic urolithin-A has a promising potency as alternative, antibiotic-independent treatment option to tackle acute campylobacteriosis by dampening the innate immune responses upon *C. jejuni* infection of the intestinal tract. The molecule can easily be applied via drinking water and is active in the intestines at low concentrations, even in the µM range. Besides the major modes of actions of oral synthetic urolithin-A application, our results further underline the important role of LOS mediated innate immune activation in *C. jejuni* induced enteritis and the feasibility of the here applied acute microbiota-depleted IL-10^−/−^ mouse model for the preclinical evaluation of future treatment options in acute campylobacteriosis. 

## Data Availability

The data presented in this study are available on request from the corresponding author.

## Supplementary Materials

The following are available online at https://www.mdpi.com/2076-0817/10/1/7/s1, Figure S1: Kinetic survey of fecal pathogen loads following urolithin-A treatment of *C. jejuni* infected IL-10^−/−^ mice. Figure S2: Kinetic survey of the clinical outcome of *C. jejuni* infected IL-10^−/−^ mice upon urolithin-A treatment.

## References

[1] Price A., Jewkes J., Sanderson P. (1979). Acute diarrhoea: *Campylobacter* colitis and the role of rectal biopsy. J. Clin. Pathol..

[2] Walker R.I., Caldwell M.B., Lee E.C., Guerry P., Trust T.J., Ruiz-Palacios G.M. (1986). Pathophysiology of *Campylobacter* enteritis. Microbiol. Rev..

[3] Moore J.E., Corcoran D., Dooley J.S., Fanning S., Lucey B., Matsuda M., McDowell D.A., Mégraud F., Millar B.C., O’Mahony R. (2005). Campylobacter. Vet. Res..

[4] Young K.T., Davis L.M., Dirita V.J. (2007). *Campylobacter jejuni*: Molecular biology and pathogenesis. Nat. Rev. Microbiol..

[5] Kaakoush N.O., Castano-Rodriguez N., Mitchell H.M., Man S.M. (2015). Global Epidemiology of *Campylobacter* Infection. Clin. Microbiol. Rev..

[6] Backert S., Tegtmeyer N., Cróinín T.Ó., Boehm M., Heimesaat M.M., Klein G. (2017). Chapter 1—Human campylobacteriosis. Campylobacter.

[7] Mousavi S., Bereswill S., Heimesaat M.M. (2020). Novel Clinical *Campylobacter jejuni* Infection Models Based on Sensitization of Mice to Lipooligosaccharide, a Major Bacterial Factor Triggering Innate Immune Responses in Human Campylobacteriosis. Microorganisms.

[8] De Zoete M.R., Keestra A.M., Roszczenko P., van Putten J.P. (2010). Activation of human and chicken toll-like receptors by *Campylobacter* spp.. Infect. Immun..

[9] Taveira da Silva A.M., Kaulbach H.C., Chuidian F.S., Lambert D.R., Suffredini A.F., Danner R.L. (1993). Brief report: Shock and multiple-organ dysfunction after self-administration of *Salmonella* endotoxin. N. Engl. J. Med..

[10] Bücker R., Krug S.M., Moos V., Bojarski C., Schweiger M.R., Kerick M., Fromm A., Janssen S., Fromm M., Hering N.A. (2018). *Campylobacter jejuni* impairs sodium transport and epithelial barrier function via cytokine release in human colon. Mucosal Immunol..

[11] Mortensen N.P., Kuijf M.L., Ang C.W., Schiellerup P., Krogfelt K.A., Jacobs B.C., van Belkum A., Endtz H.P., Bergman M.P. (2009). Sialylation of *Campylobacter jejuni* lipo-oligosaccharides is associated with severe gastro-enteritis and reactive arthritis. Microbes Infect..

[12] Bereswill S., Fischer A., Plickert R., Haag L.M., Otto B., Kuhl A.A., Dasti J.I., Zautner A.E., Munoz M., Loddenkemper C. (2011). Novel murine infection models provide deep insights into the “menage a trois” of *Campylobacter jejuni*, microbiota and host innate immunity. PLoS ONE.

[13] Warren H.S., Fitting C., Hoff E., Adib-Conquy M., Beasley-Topliffe L., Tesini B., Liang X., Valentine C., Hellman J., Hayden D. (2010). Resilience to bacterial infection: Difference between species could be due to proteins in serum. J. Infect. Dis..

[14] Mansfield L.S., Bell J.A., Wilson D.L., Murphy A.J., Elsheikha H.M., Rathinam V.A., Fierro B.R., Linz J.E., Young V.B. (2007). C57BL/6 and congenic interleukin-10-deficient mice can serve as models of *Campylobacter jejuni* colonization and enteritis. Infect. Immun..

[15] Stahl M., Ries J., Vermeulen J., Yang H., Sham H.P., Crowley S.M., Badayeva Y., Turvey S.E., Gaynor E.C., Li X. (2014). A novel mouse model of *Campylobacter jejuni* gastroenteritis reveals key pro-inflammatory and tissue protective roles for Toll-like receptor signaling during infection. PLoS Pathog..

[16] Van Spreeuwel J.P., Duursma G.C., Meijer C.J., Bax R., Rosekrans P.C., Lindeman J. (1985). *Campylobacter* colitis: Histological immunohistochemical and ultrastructural findings. Gut.

[17] Janssen R., Krogfelt K.A., Cawthraw S.A., van Pelt W., Wagenaar J.A., Owen R.J. (2008). Host-pathogen interactions in *Campylobacter* infections: The host perspective. Clin. Microbiol. Rev..

[18] Haag L.M., Fischer A., Otto B., Plickert R., Kuhl A.A., Gobel U.B., Bereswill S., Heimesaat M.M. (2012). *Campylobacter jejuni* induces acute enterocolitis in gnotobiotic IL-10-/- mice via Toll-like-receptor-2 and -4 signaling. PLoS ONE.

[19] Schmidt A.-M., Escher U., Mousavi S., Tegtmeyer N., Boehm M., Backert S., Bereswill S., Heimesaat M.M. (2019). Immunopathological properties of the *Campylobacter jejuni* flagellins and the adhesin CadF as assessed in a clinical murine infection model. Gut Pathog..

[20] Heimesaat M.M., Schmidt A.-M., Mousavi S., Escher U., Tegtmeyer N., Wessler S., Gadermaier G., Briza P., Hofreuter D., Bereswill S. (2020). Peptidase PepP is a novel virulence factor of *Campylobacter jejuni* contributing to murine campylobacteriosis. Gut Microbes.

[21] Schmidt A.M., Escher U., Mousavi S., Boehm M., Backert S., Bereswill S., Heimesaat M.M. (2019). Protease Activity of *Campylobacter jejuni* HtrA Modulates Distinct Intestinal and Systemic Immune Responses in Infected Secondary Abiotic IL-10 Deficient Mice. Front. Cell Infect. Microbiol..

[22] Heimesaat M.M., Alutis M., Grundmann U., Fischer A., Tegtmeyer N., Bohm M., Kuhl A.A., Gobel U.B., Backert S., Bereswill S. (2014). The role of serine protease HtrA in acute ulcerative enterocolitis and extra-intestinal immune responses during *Campylobacter jejuni* infection of gnotobiotic IL-10 deficient mice. Front. Cell Infect. Microbiol..

[23] Mousavi S., Escher U., Thunhorst E., Kittler S., Kehrenberg C., Bereswill S., Heimesaat M.M. (2020). Vitamin C alleviates acute enterocolitis in *Campylobacter jejuni* infected mice. Sci. Rep..

[24] Mousavi S., Lobo de Sa F.D., Schulzke J.D., Bucker R., Bereswill S., Heimesaat M.M. (2019). Vitamin D in Acute Campylobacteriosis-Results from an Intervention Study Applying a Clinical *Campylobacter jejuni* Induced Enterocolitis Model. Front. Immunol..

[25] Mousavi S., Schmidt A.-M., Escher U., Kittler S., Kehrenberg C., Thunhorst E., Bereswill S., Heimesaat M.M. (2020). Carvacrol ameliorates acute campylobacteriosis in a clinical murine infection model. Gut Pathog..

[26] Heimesaat M.M., Mousavi S., Kløve S., Genger C., Weschka D., Tamas A., Reglodi D., Bereswill S. (2020). Pituitary Adenylate Cyclase-Activating Polypeptide Alleviates Intestinal, Extra-Intestinal and Systemic Inflammatory Responses during Acute *Campylobacter jejuni*-induced Enterocolitis in Mice. Pathogens.

[27] Heimesaat M.M., Mousavi S., Kløve S., Genger C., Weschka D., Giladi E., Bereswill S., Gozes I. (2020). Immune-modulatory Properties of the Octapeptide NAP in *Campylobacter jejuni* Infected Mice Suffering from Acute Enterocolitis. Microorganisms.

[28] Johanningsmeier S.D., Harris G.K. (2011). Pomegranate as a functional food and nutraceutical source. Annu. Rev. Food Sci. Technol..

[29] Lipińska L., Klewicka E., Sójka M. (2014). The structure, occurrence and biological activity of ellagitannins: A general review. Acta Sci. Pol. Technol. Aliment..

[30] Zarfeshany A., Asgary S., Javanmard S.H. (2014). Potent health effects of pomegranate. Adv. Biomed. Res..

[31] Tomás-Barberán F.A., González-Sarrías A., García-Villalba R., Núñez-Sánchez M.A., Selma M.V., García-Conesa M.T., Espín J.C. (2017). Urolithins, the rescue of “old” metabolites to understand a “new” concept: Metabotypes as a nexus among phenolic metabolism, microbiota dysbiosis, and host health status. Mol. Nutr. Food Res..

[32] Larrosa M., González-Sarrías A., Yáñez-Gascón M.J., Selma M.V., Azorín-Ortuño M., Toti S., Tomás-Barberán F., Dolara P., Espín J.C. (2010). Anti-inflammatory properties of a pomegranate extract and its metabolite urolithin-A in a colitis rat model and the effect of colon inflammation on phenolic metabolism. J. Nutr. Biochem..

[33] Espín de Gea J.C., Larrosa M., García-Conesa M.T., Tomás Barberán F. (2013). Biological Significance of Urolithins, the Gut Microbial Ellagic Acid-Derived Metabolites: The Evidence So Far. Evid. Based Complement. Altern. Med..

[34] Cásedas G., Les F., Choya-Foces C., Hugo M., López V. (2020). The metabolite urolithin-A ameliorates oxidative stress in Neuro-2a Cells, becoming a potential neuroprotective agent. Antioxidants.

[35] Piwowarski J.P., Kiss A.K., Granica S., Moeslinger T. (2015). Urolithins, gut microbiota-derived metabolites of ellagitannins, inhibit LPS-induced inflammation in RAW 264.7 murine macrophages. Mol. Nutr. Food Res..

[36] Bobowska A., Granica S., Filipek A., Melzig M.F., Moeslinger T., Zentek J., Kruk A., Piwowarski J.P. (2020). Comparative studies of urolithins and their phase II metabolites on macrophage and neutrophil functions. Eur. J. Nutr..

[37] Djedjibegovic J., Marjanovic A., Panieri E., Saso L. (2020). Ellagic Acid-Derived Urolithins as Modulators of Oxidative Stress. Oxidative Med. Cell. Longev..

[38] Saha P., Yeoh B.S., Singh R., Chandrasekar B., Vemula P.K., Haribabu B., Vijay-Kumar M., Jala V.R. (2016). Gut microbiota conversion of dietary ellagic acid into bioactive phytoceutical urolithin A inhibits heme peroxidases. PLoS ONE.

[39] Heimesaat M.M., Bereswill S., Fischer A., Fuchs D., Struck D., Niebergall J., Jahn H.K., Dunay I.R., Moter A., Gescher D.M. (2006). Gram-negative bacteria aggravate murine small intestinal Th1-type immunopathology following oral infection with *Toxoplasma gondii*. J. Immunol..

[40] Nagayama K., Iwamura Y., Shibata T., Hirayama I., Nakamura T. (2002). Bactericidal activity of phlorotannins from the brown alga *Ecklonia kurome*. J. Antimicrob. Chemother..

[41] Nohynek L.J., Alakomi H.-L., Kähkönen M.P., Heinonen M., Helander I.M., Oksman-Caldentey K.-M., Puupponen-Pimiä R.H. (2006). Berry phenolics: Antimicrobial properties and mechanisms of action against severe human pathogens. Nutr. Cancer.

[42] Ríos J.-L., Giner R.M., Marín M., Recio M.C. (2018). A pharmacological update of ellagic acid. Planta Med..

[43] Kujawska M., Jodynis-Liebert J. (2020). Potential of the ellagic acid-derived gut microbiota metabolite–Urolithin A in gastrointestinal protection. World J. Gastroenterol..

[44] Singh R., Chandrashekharappa S., Bodduluri S.R., Baby B.V., Hegde B., Kotla N.G., Hiwale A.A., Saiyed T., Patel P., Vijay-Kumar M. (2019). Enhancement of the gut barrier integrity by a microbial metabolite through the Nrf2 pathway. Nat. Commun..

[45] Hassanein E.H., Sayed A.M., Hussein O.E., Mahmoud A.M. (2020). Coumarins as Modulators of the Keap1/Nrf2/ARE Signaling Pathway. Oxidative Med. Cell. Longev..

[46] Komatsu W., Kishi H., Yagasaki K., Ohhira S. (2018). Urolithin A attenuates pro-inflammatory mediator production by suppressing PI3-K/Akt/NF-κB and JNK/AP-1 signaling pathways in lipopolysaccharide-stimulated RAW264 macrophages: Possible involvement of NADPH oxidase-derived reactive oxygen species. Eur. J. Pharmacol..

[47] Xu J., Yuan C., Wang G., Luo J., Ma H., Xu L., Mu Y., Li Y., Seeram N.P., Huang X. (2018). Urolithins attenuate LPS-induced neuroinflammation in BV2Microglia via MAPK, Akt, and NF-κB signaling pathways. J. Agric. Food Chem..

[48] Sun X., Threadgill D., Jobin C. (2012). *Campylobacter jejuni* induces colitis through activation of mammalian target of rapamycin signaling. Gastroenterology.

[49] Totiger T.M., Srinivasan S., Jala V.R., Lamichhane P., Dosch A.R., Gaidarski A.A., Joshi C., Rangappa S., Castellanos J., Vemula P.K. (2019). Urolithin A, a novel natural compound to target PI3K/AKT/mTOR pathway in pancreatic cancer. Mol. Cancer Ther..

[50] Fu X., Gong L.-F., Wu Y.-F., Lin Z., Jiang B.-J., Wu L., Yu K.-H. (2019). Urolithin A targets the PI3K/Akt/NF-κB pathways and prevents IL-1β-induced inflammatory response in human osteoarthritis: In vitro and in vivo studies. Food Funct..

[51] Ishimoto H., Shibata M., Myojin Y., Ito H., Sugimoto Y., Tai A., Hatano T. (2011). In vivo anti-inflammatory and antioxidant properties of ellagitannin metabolite urolithin A. Bioorg. Med. Chem. Lett..

[52] Avila-Galvez M., Gimenez-Bastida J., Gonzalez-Sarrias A., Espin J. (2019). Tissue deconjugation of urolithin A glucuronide to free urolithin A in systemic inflammation. Food Funct..

[53] Heilman J., Andreux P., Tran N., Rinsch C., Blanco-Bose W. (2017). Safety assessment of Urolithin A, a metabolite produced by the human gut microbiota upon dietary intake of plant derived ellagitannins and ellagic acid. Food Chem. Toxicol..

[54] Ekmekciu I., von Klitzing E., Fiebiger U., Escher U., Neumann C., Bacher P., Scheffold A., Kühl A.A., Bereswill S., Heimesaat M.M. (2017). Immune responses to broad-spectrum antibiotic treatment and fecal microbiota transplantation in mice. Front. Immunol..

[55] Heimesaat M.M., Haag L.M., Fischer A., Otto B., Kuhl A.A., Gobel U.B., Bereswill S. (2013). Survey of extra-intestinal immune responses in asymptomatic long-term *Campylobacter jejuni*-infected mice. Eur. J. Microbiol. Immunol. (Bp).

[56] Erben U., Loddenkemper C., Doerfel K., Spieckermann S., Haller D., Heimesaat M.M., Zeitz M., Siegmund B., Kühl A.A. (2014). A guide to histomorphological evaluation of intestinal inflammation in mouse models. Int. J. Clin. Exp. Pathol..

[57] Heimesaat M.M., Giladi E., Kuhl A.A., Bereswill S., Gozes I. (2018). The octapetide NAP alleviates intestinal and extra-intestinal anti-inflammatory sequelae of acute experimental colitis. Peptides.

[58] Bryan N.S., Grisham M.B. (2007). Methods to detect nitric oxide and its metabolites in biological samples. Free Radic. Biol. Med..

