# Supplementary figures and images for: Preclinical Evaluation of Oral Urolithin-A for the Treatment of Acute Campylobacteriosis in Campylobacter jejuni Infected Microbiota-Depleted IL-10−/− Mice
Source: Pathogens. 2020 Dec 23;10(1):7. doi: 10.3390/pathogens10010007 (PMC7823290; doi:10.3390/pathogens10010007)

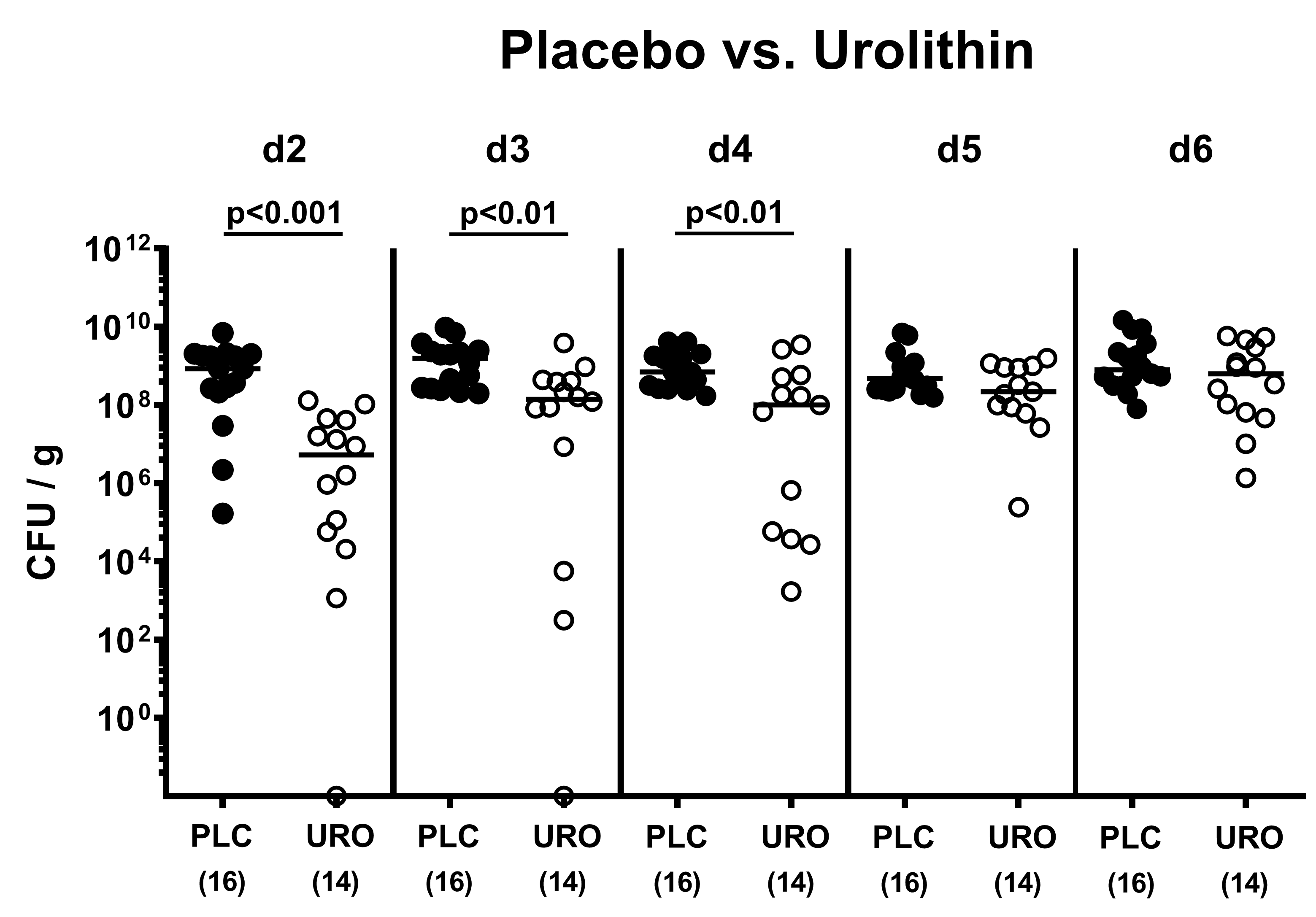

Supplement: Supplementary file 1 [file pathogens-10-00007-s001.zip › Supp_1_Placebo vs URO.tif]

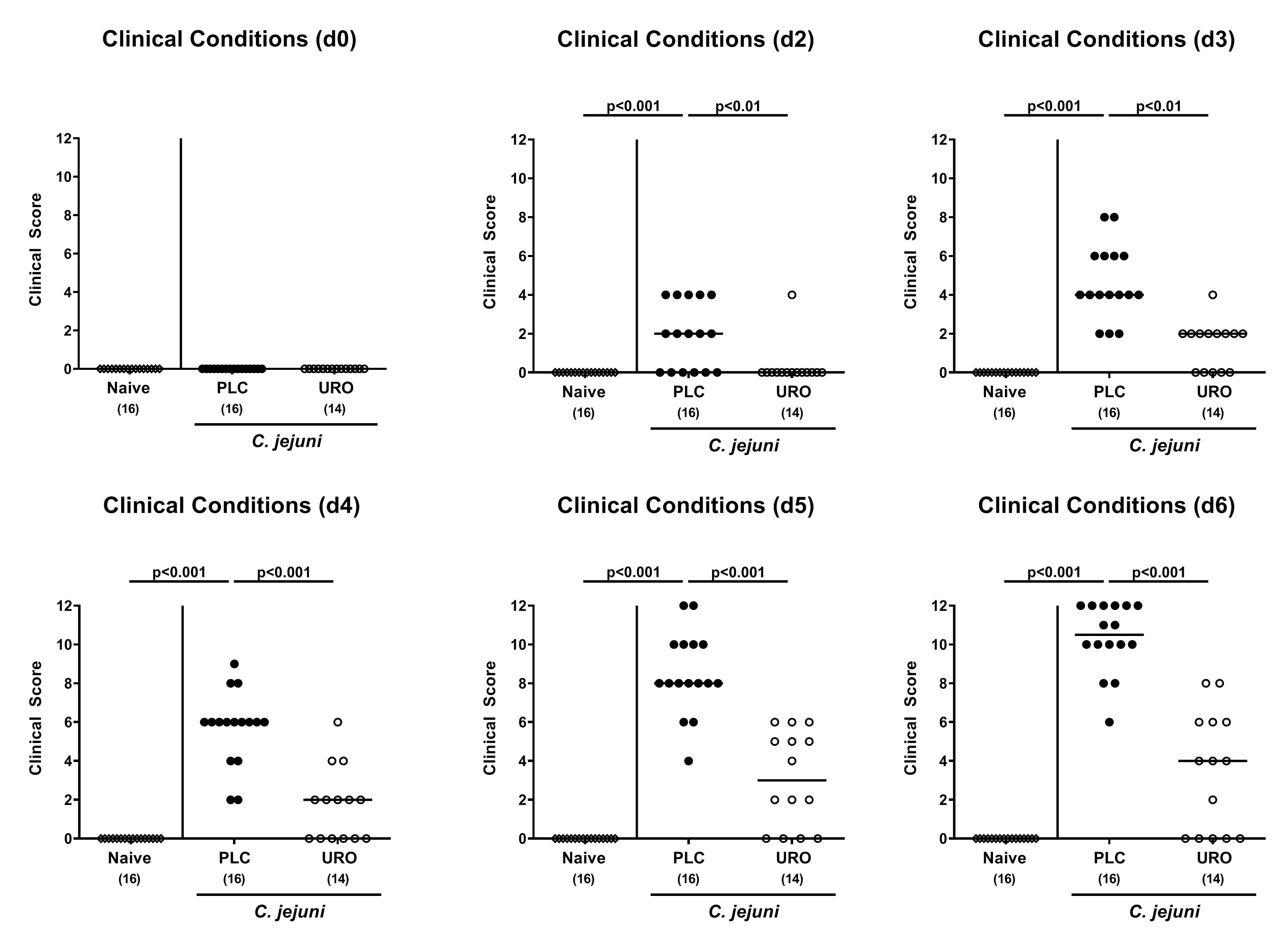

Supplement: Supplementary file 1 [file pathogens-10-00007-s001.zip › Supp_2_Urolithin_CS_d0-d6.tif]
